# Cellular and Molecular Mechanisms in Oxidative Stress-Related Diseases 2.0/3.0

**DOI:** 10.3390/ijms242116018

**Published:** 2023-11-06

**Authors:** Alessia Remigante, Rossana Morabito

**Affiliations:** Department of Chemical, Biological, Pharmaceutical and Environmental Sciences, University of Messina, 98166 Messina, Italy; rmorabito@unime.it

Oxidative stress is frequently described as the balance between the production of reactive species (including oxygen and nitrogen) in biological systems and the ability of the latter to defend itself through the sophisticated antioxidant machinery. At physiological levels, some oxidants, in controlled amounts, possess important signaling functions within the cell [1,2]. Specifically, cells can generate reactive species with the function of second messengers, using them for intracellular signaling and for stimulating the redox-sensitive signaling pathways to modify the cellular content of the cytoprotective regulatory proteins [3]. In fact, the redox state of the cell is normally regulated by a complex endogenous antioxidant system, composed of proteins with enzymatic activity and non-enzymatic proteins able to quickly neutralize, or ensure a low production of, reactive species [4]. Nevertheless, when oxidants are produced in excess, or when the antioxidant defenses that regulate them are ineffective, this balance can be perturbed, thus resulting in an oxidative condition. Oxidative products are highly reactive, and can directly or indirectly modulate the functions of many enzymes and transcription factors through complex signaling cascades. In particular, some of the pathways are preferentially linked to enhanced survival, while others are more frequently associated with cell death, and constitute important avenues for therapeutic interventions aimed at limiting oxidative damage or, alternatively, attenuating its consequences [5,6,7,8,9,10,11]. Furthermore, the magnitude and exposure of the insult, as well as the cell type involved, are key elements in defining which pathways are activated, as well as the final cell outcome. This Special Issue has been conceived to collect and contribute to the dissemination of novel findings unraveling the impact of oxidative stress on cells, their subcellular components, and biological macromolecules. Here, we offer an overview of the content of this Special Issue, which collects 13 original articles and seven reviews.

## 1. Original Articles

(1) “Polymorphisms in Genes Encoding VDR, CALCR and Antioxidant Enzymes as Predictors of Bone Tissue Condition in Young, Healthy Men” by Jowko, E. and collaborators [12]. The aim of the study was to assess significant predictors of bone mineral content (BMC) and bone mineral density (BMD) in a group of young, healthy men at the time of reaching peak bone mass. Regression analyses showed that age, BMI, and practicing combat and team sports at a competitive level (trained vs. untrained group; TR vs. CON, respectively) were positive predictors of BMD/BMC values at various skeletal sites. In addition, genetic polymorphisms were among the predictors. In the whole population studied, at almost all measured skeletal sites, the SOD2 AG genotype proved to be a negative predictor of BMC, while the VDR FokI GG genotype was a negative predictor of BMD. In contrast, the CALCR AG genotype was a positive predictor of arm BMD. ANOVA analyses showed that, regarding the SOD2 polymorphism, the TR group was responsible for the significant intergenotypic differences in BMC that were observed in the whole study population (i.e., lower BMC values of leg, trunk, and whole body were observed in AG TR compared to AA TR). On the other hand, a higher BMC at L1–L4 was observed in the SOD2 GG genotype of the TR group compared to in the same genotype of the CON group. For the FokI polymorphism, the BMD at L1–L4 was higher in AG TR than in AG CON. In turn, the CALCR AA genotype in the TR group had higher arm BMD compared to the same genotype in the CON group. In conclusion, SOD2, VDR FokI, and CALCR polymorphisms seem to affect the association of BMC/BMD values with training status. In general, at least within the VDR FokI and CALCR polymorphisms, less favorable genotypes in terms of BMD (i.e., FokI AG and CALCR AA) appear to be associated with a greater BMD response to sports training. This suggests that, in healthy men during the period of bone mass formation, sports training (combat and team sports) may attenuate the negative impact of genetic factors on bone tissue condition, possibly reducing the risk of osteoporosis in later age.
(2) “Usefulness of Urinary Biomarkers for Assessing Bladder Condition and Histopathology in Patients with Interstitial Cystitis/Bladder Pain Syndrome” by Jiang, Y. H. and collaborators [13]. This study investigated the usefulness of urinary biomarkers for assessing the bladder condition and histopathology in patients with interstitial cystitis/bladder pain syndrome (IC/BPS). We retrospectively enrolled 315 patients (267 women and 48 men) diagnosed with IC/BPS and 30 controls. Data on clinical and urodynamic characteristics (visual analog scale (VAS) score and bladder capacity) and cystoscopic hydrodistention findings (Hunner’s lesion, glomerulation grade, and maximal bladder capacity (MBC)) were recorded. Urine samples were utilized to assay inflammatory, neurogenic, and oxidative stress biomarkers, including interleukin (IL)-8, C-X-C motif chemokine ligand 10 (CXCL10), monocyte chemoattractant protein-1 (MCP-1), brain-derived neurotrophic factor (BDNF), eotaxin, IL-6, macrophage inflammatory protein 1 beta (MIP-1β), regulated on activation, normal T cell expressed and secreted (RANTES), tumor necrosis factor-alpha (TNF-α), prostaglandin E2 (PGE2), 8-hydroxy-2′-deoxyguanosine (8-OHdG), 8-isoproatane, and total antioxidant capacity. Further, specific histopathological findings were identified via bladder biopsies. The associations between urinary biomarker levels, bladder conditions, and histopathological findings were evaluated. The results reveal that patients with IC/BPS had significantly higher urinary MCP-1, eotaxin, TNF-α, PGE2, 8-OHdG, and 8-isoprostane levels than the controls. Patients with Hunner’s IC (HIC) had significantly higher IL-8, CXCL10, BDNF, eotaxin, IL-6, MIP-1β, and RANTES levels than those with non-Hunner’s IC (NHIC). Patients with NHIC who had an MBC of ≤760 mL had significantly high urinary CXCL10, MCP-1, eotaxin, IL-6, MIP-1β, RANTES, PGE2, and 8-isoprostane levels and total antioxidant capacity. Patients with NHIC who had a higher glomerulation grade had significantly high urinary MCP-1, IL-6, RANTES, 8-OHdG, and 8-isoprostane levels. A significant association was observed between urinary biomarkers and glomerulation grade, MBC, VAS score, and bladder sensation. However, bladder-specific histopathological findings were not well correlated with urinary biomarker levels. The urinary biomarker levels can be useful for identifying HIC and different NHIC subtypes. Higher urinary inflammatory and oxidative stress biomarker levels are associated with IC/BPS. Most urinary biomarkers are not correlated with specific bladder histopathological findings; nevertheless, they are more important in the assessment of bladder condition than in bladder histopathology.
(3) “Endoplasmic Reticulum Stress Promotes the Expression of TNF-α in THP-1 Cells by Mechanisms Involving ROS/CHOP/HIF-1α and MAPK/NF-κB Pathways” by Akhter, N. and collaborators [14]. Obesity and metabolic syndrome involve chronic low-grade inflammation, called metabolic inflammation, as well as metabolic derangements from increased endotoxin and free fatty acids. It is debated whether the endoplasmic reticulum (ER) stress in monocytic cells can contribute to amplify metabolic inflammation and, if so, by which mechanism(s). To test this, metabolic stress was induced in THP-1 cells and primary human monocytes by treatments with lipopolysaccharide (LPS), palmitic acid (PA), or oleic acid (OA), in the presence or absence of the ER stressor thapsigargin (TG). Gene expression of tumor necrosis factor (*TNF*)-*α* and markers of ER/oxidative stress were determined by qRT-PCR, TNF-α protein by ELISA, reactive oxygen species (ROS) by DCFH-DA assay, hypoxia-inducible factor 1-alpha (HIF-1α), p38, extracellular signal-regulated kinase (ERK)-1,2, and nuclear factor kappa B (NF-κB) phosphorylation by immunoblotting, and insulin sensitivity by glucose-uptake assay. Regarding clinical analyses, adipose TNF-α was assessed using qRT-PCR/IHC and plasma TNF-α, high-sensitivity C-reactive protein (hs-CRP), malondialdehyde (MDA), and oxidized low-density lipoprotein (OX-LDL) via ELISA. We found that the cooperative interaction between metabolic and ER stresses promoted TNF-α, ROS, CCAAT-enhancer-binding protein homologous protein (*CHOP*), activating transcription factor 6 (*ATF6*), superoxide dismutase 2 (*SOD2*), and nuclear factor erythroid 2-related factor 2 (*NRF2*) expression (*p* ≤ 0.0183). However, glucose uptake was not impaired. TNF-α amplification was dependent on HIF-1α stabilization and p38 MAPK/p65 NF-κB phosphorylation, while the MAPK/NF-κB pathway inhibitors and antioxidants/ROS scavengers, such as curcumin, allopurinol, and apocynin, attenuated the TNF-α production (*p* ≤ 0.05). Individuals with obesity displayed increased adipose TNF-α gene/protein expression, as well as elevated plasma levels of TNF-α, CRP, MDA, and OX-LDL (*p* ≤ 0.05). Our findings support a metabolic–ER stress cooperativity model, favoring inflammation by triggering TNF-α production via the ROS/CHOP/HIF-1α and MAPK/NF-κB dependent mechanisms. This study also highlights the therapeutic potential of antioxidants in inflammatory conditions involving metabolic/ER stresses.
(4) “Impact of Truncated Oxidized Phosphatidylcholines on Phospholipase A2 Activity in Mono- and Polyunsaturated Biomimetic Vesicles” by Yordanova, V. and collaborators [15]. The interplay between inflammatory and redox processes is a ubiquitous and critical phenomenon in cell biology that involves numerous biological factors. Among them, secretory phospholipases A2 (sPLA2), which catalyze the hydrolysis of the sn-2 ester bond of phospholipids, are key players. They can interact or be modulated by the presence of truncated oxidized phosphatidylcholines (OxPCs) produced under oxidative stress from phosphatidylcholine (PC) species. The present study examined this important, but rarely considered, sPLA2 modulation induced by changes in the biophysical properties of PC vesicles comprising various OxPC ratios in mono- or poly-unsaturated PCs. Being the most physiologically active OxPCs, 1-palmitoyl-2-(5′-oxo-valeroyl)-sn-glycero-3-phosphocholine (POVPC) and 1-palmitoyl-2-glutaryl-sn-glycero-3-phosphocholine (PGPC) have been selected for this study. Using fluorescence spectroscopy methods, we compared the effect of OxPCs on the lipid order and sPLA2 activity in large unilamellar vesicles (LUVs) made of the heteroacid PC, either monounsaturated (1-palmitoyl-2-oleoyl-sn-glycero-3-phosphocholine (POPC)) or polyunsaturated (1-palmitoyl-2-docosahexaenoyl-sn-glycero-3-phosphocholine (PDPC)), at a physiological temperature. The effect of OxPCs on vesicle size was also assessed in both the mono- and polyunsaturated PC matrices. The results showed that OxPCs decrease the membrane lipid order of POPC and PDPC mixtures with PGPC, inducing a much larger decrease in comparison with POVPC, indicative that the difference takes place at the glycerol level. Compared to POPC, PDPC was able to inhibit sPLA2 activity, showing a protective effect of PDPC against enzyme hydrolysis. Furthermore, sPLA2 activity on its PC substrates was modulated by the OxPC membrane content. POVPC down-regulated sPLA2 activity, suggesting anti-inflammatory properties of this truncated oxidized lipid. Interestingly, PGPC had a dual and opposite effect, either inhibiting or enhancing sPLA2 activity, depending on the protocol of lipid mixing. This difference may result from the chemical properties of the shortened sn-2-acyl chain residues (aldehyde group for POVPC, and carboxyl for PGPC), being, respectively, zwitterionic or anionic under hydration at physiological conditions.
(5) “Electrophoretic Determination of L-Carnosine in Health Supplements Using an Integrated Lab-on-a-Chip Platform with Contactless Conductivity Detection” by Pukleš, I. and collaborators [16]. The health supplement industry is one of the fastest growing industries in the world, but there is a lack of suitable analytical methods for the determination of active compounds in health supplements, such as peptides. The present work describes an implementation of contactless conductivity detection on microchip technology as a new strategy for the electrophoretic determination of L-carnosine in complex health supplement formulations without pre-concentration and derivatization steps. The best results were obtained in the case of +1.00 kV applied for 20 s for injection and +2.75 kV applied for 260 s for the separation step. Under the selected conditions, a linear detector response of 5 × 10^−6^ to 5 × 10^−5^ M was achieved. L-carnosine retention time was 61 s. The excellent reproducibility of both migration time and detector response confirmed the high precision of the method. The applicability of the method was demonstrated by the determination of L-carnosine in three different samples of health supplements. The recoveries ranged from 91 to 105%. Subsequent analyses of the samples by CE-UV-VIS and HPLC-DAD confirmed the accuracy of the obtained results.
(6) “Evaluation of Oxidative Stress and Metabolic Profile in a Preclinical Kidney Transplantation Model According to Different Preservation Modalities” by Mrakic-Sposta and collaborators [17]. This study addresses a joint nuclear magnetic resonance (NMR) and electron paramagnetic resonance (EPR) spectroscopy approach to providing a platform for the dynamic assessment of kidney viability and metabolism. On porcine kidney models, ROS production, oxidative damage kinetics, and metabolic changes occurring both during the period between organ retrieval and im- plantation and after kidney graft were examined. The ^1^H-NMR metabolic profile-valine, alanine, acetate, trimetylamine-N-oxide, glutathione, lactate, and the EPR oxidative stress resulting from ischemia/reperfusion injury after preservation (8 h) by static cold storage (SCS) and ex vivo machine perfusion (HMP) methods were monitored. The functional recovery after transplantation (14 days) was evaluated by serum creatinine (SCr), oxidative stress (ROS), and damage (thiobarbituric-acid reactive substances and protein carbonyl enzymatic) assessments. At 8 h of preservation storage, a significantly (*p* < 0.0001) higher ROS production was measured in the SCS group, as compared to the HMP group. Significantly higher concentration data (*p* < 0.05–0.0001) in HMP vs. SCS for all the monitored metabolites were found as well. The HMP group showed a better function recovery. The comparison of the areas under the SCr curves (AUCs) returned a significantly smaller (−12.5%) AUC in the HMP vs. SCS. The EPR-ROS concentrations (μmol·g^−1^) from bioptic kidney tissue samples were significantly lower in HMP vs. SCS. The same result was found for the NMR monitored metabolites: lactate: −59.76%, alanine: −43.17%; valine: −58.56%; and TMAO: −77.96%. No changes were observed in either group under light microscopy. In conclusion, a better and more rapid normalization of oxidative stress and functional recovery after transplantation were observed by HMP utilization.
(7) “Protective Effect of Resveratrol in an Experimental Model of Salicylate-Induced Tinnitus” by Song, A. and collaborators [18]. To date, the effect of resveratrol on tinnitus has not been reported. The attenuative effects of resveratrol (RSV) on a salicylate-induced tinnitus model were evaluated by in vitro and in vivo experiments. The gene expression of the activity-regulated cytoskeleton-associated protein (*ARC*), tumor necrosis factor-alpha (TNF-α), and NMDA receptor subunit 2B (*NR2B*) in SH-SY5Y cells was examined using qPCR. Phosphorylated cAMP response element-binding protein (p-CREB), apoptosis markers, and reactive oxygen species (ROS) were evaluated by in vitro experiments. The in vivo experiment evaluated the gap-prepulse inhibition of the acoustic startle reflex (GPIAS) and auditory brainstem response (ABR) level. The *NR2B* expression in the auditory cortex (AC) was determined by immunohistochemistry. RSV significantly reduced the salicylate-induced expression of *NR2B*, *ARC*, and TNF-α in neuronal cells; the GPIAS and ABR thresholds altered by salicylate in rats were recovered close to their normal range. RSV also reduced the salicylate-induced NR2B overexpression of the AC. These results confirmed that resveratrol exerted an attenuative effect on salicylate-induced tinnitus, and may have therapeutic potential.
(8) “Molecular Mechanisms of Oxidative Stress Relief by CAPE in ARPE−19 Cells” by Ren, C. and collaborators [19]. Caffeic acid phenylethyl ester (CAPE) is an antioxidative agent originally derived from propolis. Oxidative stress is a significant pathogenic factor in most retinal diseases. Our previous study revealed that CAPE suppresses mitochondrial ROS production in ARPE−19 cells by regulating UCP2. The present study explores the ability of CAPE to provide longer-term protection to RPE cells and the underlying signal pathways involved. ARPE−19 cells were given CAPE pretreatment, followed by t-BHP stimulation. We used in situ live cell staining with CellROX and MitoSOX to measure ROS accumulation; an Annexin V-FITC/PI assay to evaluate cell apoptosis; ZO−1 immunostaining to observe the tight junction integrity in the cells; RNA-seq to analyze changes in gene expression; q-PCR to validate the RNA-seq data; and a Western blot to examine MAPK signal pathway activation. CAPE significantly reduced both cellular and mitochondria ROS overproduction, restored the loss of ZO−1 expression, and inhibited apoptosis induced by t-BHP stimulation. We also demonstrated that CAPE reverses the overexpression of immediate early genes (IEGs) and the activation of the p38-MAPK/CREB signal pathway. Either genetic or chemical deletion of UCP2 largely abolished the protective effects of CAPE. CAPE restrained ROS generation and preserved the tight junction structure of ARPE−19 cells against oxidative-stress-induced apoptosis. These effects were mediated via UCP2 regulation of p38/MAPK-CREB-IEGs pathway.
(9) “The Critical Assessment of Oxidative Stress Parameters as Potential Biomarkers of Carbon Monoxide Poisoning” by Hydzik, P. and collaborators [20]. In conventional clinical toxicology practice, the blood level of carboxyhemoglobin is a biomarker of carbon monoxide (CO) poisoning, but does not correspond to the complete clinical picture and the severity of the poisoning. Taking into account articles suggesting the relationship between oxidative stress parameters and CO poisoning, it seems reasonable to consider this topic more broadly, including experimental biochemical data (oxidative stress parameters) and patients poisoned with CO. This article aimed to critically assess oxidative-stress-related parameters as potential biomarkers to evaluate the severity of CO poisoning, and their possible role in the decision to treat. The critically set parameters were antioxidative, including catalase, 2,2-diphenyl-1-picryl-hydrazyl, glutathione, thiol, and carbonyl groups. The preliminary studies involved patients (*n* = 82) admitted to the Toxicology Clinical Department of the University Hospital of Jagiellonian University Medical College (Krakόw, Poland) during 2015–2020. The poisoning was diagnosed based on medical history, clinical symptoms, and carboxyhemoglobin blood level. Blood samples for carboxyhemoglobin and antioxidative parameters were collected immediately after admission to the emergency department. To evaluate the severity of the poisoning, the Pach scale was applied. The final analysis included a significant decrease in catalase activity and a reduction in glutathione level in all poisoned patients based on the severity of the Pach scale (I°–III°) compared to the control group. It follows from the experimental data that the poisoned patients had a significant increase in level due to thiol groups and the 2,2-diphenyl-1-picryl-hydrazyl radical, with no significant differences according to the severity of poisoning. The catalase-to-glutathione and thiol-to-glutathione ratios showed the most important differences between the poisoned patients and the control group, with a significant increase in the poisoned group. The ratios did not differentiate the severity of the poisoning. The carbonyl level was highest in the control group compared to the poisoned group, but was not statistically significant. Our critical assessment shows that using oxidative-stress-related parameters to evaluate the severity of CO poisoning, the outcome, and treatment options is challenging.
(10) “Microplastics Exacerbate Cadmium-Induced Kidney Injury by Enhancing Oxidative Stress, Autophagy, Apoptosis, and Fibrosis” by Zou, H. and collaborators [21]. Cadmium (Cd) is a potential pathogenic factor in the urinary system that is associated with various kidney diseases. Microplastics (MPs), comprising of plastic particles less than 5 mm in diameter, are a major carrier of contaminants. We applied 10 mg/L particle 5 μm MPs and 50 mg/L CdCl2 in water for a three month in vivo assay to assess the damaging effects of MPs and Cd exposure on the kidneys. In vivo tests showed that MPs exacerbated Cd-induced kidney injury. In addition, the involvement of oxidative stress, autophagy, apoptosis, and fibrosis in the damaging effects of MPs and Cd on mouse kidneys were investigated. The results showed that MPs aggravated Cd-induced kidney injury by enhancing oxidative stress, autophagy, apoptosis, and fibrosis. These findings provide new insights into the toxic effects of MPs on mouse kidneys.
(11) “Toxicity of Metal Ions Released from a Fixed Orthodontic Appliance to Gastrointestinal Tract Cell Lines” by Durgo, K. and collaborators [22]. The mechanisms of toxicity and cellular responses to metal ions present in the environment are still a very current area of research. In this work, which is a continuation of the study of the toxicity of metal ions released by fixed orthodontic appliances, eluates of archwires, brackets, ligatures, and bands are used to test the prooxidant effect, cytotoxicity, and genotoxicity on cell lines of the gastrointestinal tract. Eluates obtained after three immersion periods (3, 7, and 14 days) and with known amounts and types of metal ions were used. Four cell lines—CAL 27 (human tongue), Hep-G2 (liver), AGS (stomach), and CaCo-2 (colon)—were treated with each type of eluate at four concentrations (0.1×, 0.5×, 1.0×, and 2.0×) for 24 h. Most eluates had toxic effects on CAL 27 cells over the entire concentration range, regardless of exposure time, while CaCo-2 proved to be the most resistant. In AGS and Hep-G2 cells, all samples tested induced free radical formation, with the highest concentration (2×) causing a decrease in free radicals formed, compared to the lowest concentrations. Eluates containing Cr, Mn, and Al showed a slight pro-oxidant effect on DNA (on plasmid φX-174 RF I) and slight genotoxicity (comet assay), but these effects are not so great that the human body could not “resist” them. Statistical analyses of the data on chemical composition, cytotoxicity, ROS, genotoxicity, and prooxidative DNA damage shows the influence of metal ions present in some eluates on the toxicity obtained. Fe and Ni are responsible for the production of ROS, while Mn and Cr have a great influence on hydroxyl radicals, which cause single-strand breaks in supercoiled plasmid DNA, in addition to the production of ROS. On the other hand, Fe, Cr, Mn, and Al are responsible for the cytotoxic effect of the studied eluates. The obtained results confirm that this type of research is useful and brings us closer to more accurate in vivo conditions.
(12) “Lead Exposure Causes Spinal Curvature during Embryonic Development in Zebrafish” by Li, X. and collaborators [23]. Lead (Pb^2+^) is an important raw material for modern industrial production, which enters the aquatic environment in several ways and cause serious harm to aquatic ecosystems. Lead ions (Pb^2+^) are highly toxic, and can accumulate continuously in organisms. In addition to causing biological deaths, it can also cause neurological damage in vertebrates. Our experiment found that Pb^2+^ caused decreased survival, delayed hatching, decreased the frequency of voluntary movements at 24 hpf, increased the heart rate at 48 hpf, and increased the malformation rate in zebrafish embryos. Among them, the morphology of spinal malformations varied, with 0.4 mg/L Pb^2+^ causing a dorsal bending of the spine of 72 hpf zebrafish and a ventral bending in 120 hpf zebrafish. It was determined that spinal malformations were mainly caused by Pb-induced endoplasmic reticulum stress and apoptosis. The genetic changes in somatic segment development, which disrupted developmental polarity as well as osteogenesis, resulted in uneven myotomal development. In contrast, calcium ions can rescue the series of responses induced by lead exposure and reduce the occurrence of spinal curvature. This article proposes new findings for lead pollution toxicity in zebrafish.
(13) “Anti-*Candida albicans* Effects and Mechanisms of Theasaponin E1 and Assamsaponin A” by Chen, Y. and collaborators [24]. *Candida albicans* is an opportunistic human fungal pathogen, and its drug resistance is becoming a serious problem. *Camellia sinensis* seed saponins showed inhibitory effects on resistant *Candida albicans* strains, but the active components and mechanisms are unclear. In this study, the effects and mechanisms of two *Camellia sinensis* seed saponin monomers, theasaponin E1 (TE1) and assamsaponin A (ASA), on a resistant *Candida albicans* strain (ATCC 10231) were explored. The minimum inhibitory concentration and minimum fungicidal concentration of TE1 and ASA were equivalent. The time–kill curves showed that the fungicidal efficiency of ASA was higher than that of TE1. TE1 and ASA significantly increased the cell membrane permeability and disrupted the cell membrane integrity of *C. albicans* cells, probably by interacting with membrane-bound sterols. Moreover, TE1 and ASA induced the accumulation of intracellular ROSs and decreased the mitochondrial membrane potential. Transcriptome and qRT-PCR analyses revealed that the differentially expressed genes were concentrated in the cell wall, plasma membrane, glycolysis, and ergosterol synthesis pathways. In conclusion, the antifungal mechanisms of TE1 and ASA included the interference with the biosynthesis of ergosterol in fungal cell membranes, damage to the mitochondria, and the regulation of energy metabolism and lipid metabolism. Tea seed saponins have the potential to be novel anti-*Candida albicans* agents.

## 2. Reviews

(1) “Significance of Singlet Oxygen Molecule in Pathologies” by Murotomi, K. and collaborators [25]. Reactive oxygen species, including singlet oxygen, play an important role in the onset and progression of disease, as well as in aging. Singlet oxygen can be formed non-enzymatically by chemical, photochemical, and electron transfer reactions, or as a byproduct of endogenous enzymatic reactions in phagocytosis during inflammation. The imbalance of antioxidant enzymes and antioxidant networks with the generation of singlet oxygen increases oxidative stress, resulting in the undesirable oxidation and modification of biomolecules, such as proteins, DNA, and lipids. This review describes the molecular mechanisms of singlet oxygen production in vivo and methods for the evaluation of damage induced by singlet oxygen. The involvement of singlet oxygen in the pathogenesis of skin and eye diseases is also discussed from the biomolecular perspective. We also present our findings on lipid oxidation products derived from singlet oxygen-mediated oxidation in glaucoma, early diabetes patients, and a mouse model of bronchial asthma. Even in these diseases, oxidation products, due to singlet oxygen, have not been measured clinically. This review discusses their potential as biomarkers for diagnosis. Recent developments in singlet oxygen scavengers, such as carotenoids, which can be utilized to prevent the onset and progression of disease, are also described.
(2) “Manipulation of Oxidative Stress Responses by Non-Thermal Plasma to Treat Herpes Simplex Virus Type 1 Infection and Disease” by Sutter, J. and collaborators [26]. Herpes simplex virus type 1 (HSV-1) is a contagious pathogen with a large global footprint, due to its ability to cause lifelong infection in patients. Current antiviral therapies are effective in limiting viral replication in the epithelial cells to alleviate clinical symptoms, but ineffective in eliminating latent viral reservoirs in neurons. Much of HSV-1 pathogenesis is dependent on its ability to manipulate oxidative stress responses to craft a cellular environment that favors HSV-1 replication. However, to maintain redox homeostasis and to promote antiviral immune responses, the infected cell can upregulate reactive oxygen and nitrogen species (RONS) while having a tight control on antioxidant concentrations to prevent cellular damage. Non-thermal plasma (NTP), which we propose as a potential therapy alternative directed against HSV-1 infection, is a means to deliver RONS that affect redox homeostasis in the infected cell. This review emphasizes how NTP can be an effective therapy for HSV-1 infections through the direct antiviral activity of RONS and via immunomodulatory changes in the infected cells that will stimulate anti-HSV-1 adaptive immune responses. Overall, NTP applications can control HSV-1 replication and address the challenges of latency by decreasing the size of the viral reservoir in the nervous system.
(3) “Recent Developments in the Understanding of Immunity, Pathogenesis and Management of COVID-19” by Yegiazaryan, A. and collaborators [27]. Coronaviruses represent a diverse family of enveloped positive-sense single stranded RNA viruses. COVID-19, caused by Severe Acute Respiratory Syndrome Coronavirus-2, is a highly contagious respiratory disease transmissible mainly via close contact and respiratory droplets, which can result in severe, life-threatening respiratory pathologies. It is understood that glutathione, a naturally occurring antioxidant known for its role in immune response and cellular detoxification, is the target of various proinflammatory cytokines and transcription factors resulting in the infection, replication, and production of reactive oxygen species. This leads to more severe symptoms of COVID-19 and increased susceptibility to other illnesses, such as tuberculosis. The emergence of vaccines against COVID-19, the usage of monoclonal antibodies as treatments for infection, and the implementation of pharmaceutical drugs, have been effective methods for preventing and treating symptoms. However, with the mutating nature of the virus, other treatment modalities have been studied. With its role in antiviral defense and immune response, glutathione has been heavily explored in regard to COVID-19. Glutathione has demonstrated protective effects on inflammation and downregulation of reactive oxygen species, thereby resulting in less severe symptoms of COVID-19 infection and warranting the discussion of glutathione as a treatment mechanism.
(4) “The Yin and Yang Effect of the Apelinergic System in Oxidative Stress” by Fibbi, B. and collaborators [28]. Apelin is an endogenous ligand for the G protein-coupled receptor APJ and has multiple biological activities in human tissues and organs, including the heart, blood vessels, adipose tissue, central nervous system, lungs, kidneys, and liver. This article reviews the crucial role of apelin in regulating oxidative-stress-related processes by promoting prooxidant or antioxidant mechanisms. Following the binding of APJ to different active apelin isoforms and the interaction with several G proteins according to cell types, the apelin/APJ system is able to modulate different intracellular signaling pathways and biological functions, such as vascular tone, platelet aggregation and leukocytes adhesion, myocardial activity, ischemia/reperfusion injury, insulin resistance, inflammation, and cell proliferation and invasion. As a consequence of these multifaceted properties, the role of the apelinergic axis in the pathogenesis of degenerative and proliferative conditions (e.g., Alzheimer’s and Parkinson’s diseases, osteoporosis, and cancer) is currently investigated. In this view, the dual effect of the apelin/APJ system in the regulation of oxidative stress needs to be more extensively clarified, in order to identify new potential strategies and tools able to selectively modulate this axis according to the tissue-specific profile.
(5) “Molecular Genetics of Abnormal Redox Homeostasis in Type 2 Diabetes Mellitus” by Azarova, I. and collaborators [29]. Numerous studies have shown that oxidative stress resulting from an imbalance between the production of free radicals and their neutralization by antioxidant enzymes is one of the major pathological disorders underlying the development and progression of type 2 diabetes (T2D). This review summarizes the current state-of-the-art advances in understanding the role of abnormal redox homeostasis in the molecular mechanisms of T2D, and provides comprehensive information on the characteristics and biological functions of antioxidant and oxidative enzymes, as well as discussing genetic studies conducted so far in order to investigate the contribution of polymorphisms in genes encoding redox state-regulating enzymes to the disease pathogenesis.
(6) “Molecular Pathology, Oxidative Stress, and Biomarkers in Obstructive Sleep Apnea” by Meliante, P. G. and collaborators [30]. Obstructive sleep apnea syndrome (OSAS) is characterized by intermittent hypoxia (IH) during sleep due to recurrent upper airway obstruction. The derived oxidative stress (OS) leads to complications that do not only concern the sleep–wake rhythm, but also systemic dysfunctions. The aim of this narrative literature review is to investigate molecular alterations, diagnostic markers, and potential medical therapies for OSAS. We analyzed the literature and synthesized the evidence collected. IH increases oxygen free radicals (ROS) and reduces antioxidant capacities. OS and metabolic alterations lead OSAS patients to undergo endothelial dysfunction, osteoporosis, systemic inflammation, increased cardiovascular risk, pulmonary remodeling, and neurological alterations. We treated molecular alterations known to date as useful for understanding the pathogenetic mechanisms and for their potential application as diagnostic markers. The most promising pharmacological therapies are those based on N-acetylcysteine (NAC), vitamin C, leptin, dronabinol, or atomoxetine + oxybutynin, but all require further experimentation. CPAP remains the approved therapy capable of reversing most of the known molecular alterations; future drugs may be useful in treating the remaining dysfunctions.
(7) “The Potential of Cylindromatosis (CYLD) as a Therapeutic Target in Oxidative Stress-Associated Pathologies: A Comprehensive Evaluation” by Zhenzhou, H. and collaborators [31]. Oxidative stress (OS) arises as a consequence of an imbalance between the formation of reactive oxygen species (ROS) and the capacity of antioxidant defense mechanisms to neutralize them. Excessive ROS production can lead to the damage of critical biomolecules, such as lipids, proteins, and DNA, ultimately contributing to the onset and progression of a multitude of diseases, including atherosclerosis, chronic obstructive pulmonary disease, Alzheimer’s disease, and cancer. Cylindromatosis (CYLD), initially identified as a gene linked to familial cylindromatosis, has a well-established and increasingly well-characterized function in tumor inhibition and anti-inflammatory processes. Nevertheless, burgeoning evidence suggests that CYLD, as a conserved deubiquitination enzyme, also plays a pivotal role in various key signaling pathways, and is implicated in the pathogenesis of numerous diseases driven by oxidative stress. In this review, we systematically examine the current research on the function and pathogenesis of CYLD in diseases instigated by oxidative stress. Therapeutic interventions targeting CYLD may hold significant promise for the treatment and management of oxidative stress-induced human diseases.

In conclusion, the publications collected are well representative of the variety of consequences due increased oxidative stress and highlight the multitude of the experimental models and approaches currently used to unravel the potential harmful effects. We hope that this editorial effort will contribute to fueling the burgeoning field of cells and tissues responses to oxidant stress, an area we believe to be relevant not only to human physiopathology, but also medical investigations at large. Thus, the precise understanding of oxidative-stress-related disease pathophysiology could favor the identification of novel therapeutic targets, as well as antioxidant strategies.
